# Chitosan, chitosan derivatives, and chitosan-based nanocomposites: eco-friendly materials for advanced applications (a review)

**DOI:** 10.3389/fchem.2023.1327426

**Published:** 2024-01-04

**Authors:** Abir El-Araby, Walid Janati, Riaz Ullah, Sezai Ercisli, Faouzi Errachidi

**Affiliations:** ^1^ Functional Ecology and Environment Engineering Laboratory, Faculty of Science and Technology, Sidi Mohamed Ben Abdellah University, Fez, Morocco; ^2^ Medicinal Aromatic and Poisonous Plants Research Centre, College of Pharmacy, King Saud University, Riyadh, Saudi Arabia; ^3^ Department of Horticulture, Faculty of Horticulture, Ataturk University, Erzurum, Türkiye; ^4^ HGF Agro, Ata Teknokent, Erzurum, Türkiye

**Keywords:** chitosan and its derivatives, chitosan-based nanocomposites, engineering applications, functional properties, physicochemical properties

## Abstract

For many years, chitosan has been widely regarded as a promising eco-friendly polymer thanks to its renewability, biocompatibility, biodegradability, non-toxicity, and ease of modification, giving it enormous potential for future development. As a cationic polysaccharide, chitosan exhibits specific physicochemical, biological, and mechanical properties that depend on factors such as its molecular weight and degree of deacetylation. Recently, there has been renewed interest surrounding chitosan derivatives and chitosan-based nanocomposites. This heightened attention is driven by the pursuit of enhancing efficiency and expanding the spectrum of chitosan applications. Chitosan’s adaptability and unique properties make it a game-changer, promising significant contributions to industries ranging from healthcare to environmental remediation. This review presents an up-to-date overview of chitosan production sources and extraction methods, focusing on chitosan’s physicochemical properties, including molecular weight, degree of deacetylation and solubility, as well as its antibacterial, antifungal and antioxidant activities. In addition, we highlight the advantages of chitosan derivatives and biopolymer modification methods, with recent advances in the preparation of chitosan-based nanocomposites. Finally, the versatile applications of chitosan, whether in its native state, derived or incorporated into nanocomposites in various fields, such as the food industry, agriculture, the cosmetics industry, the pharmaceutical industry, medicine, and wastewater treatment, were discussed.

## 1 Introduction

**FIGURE 1:**
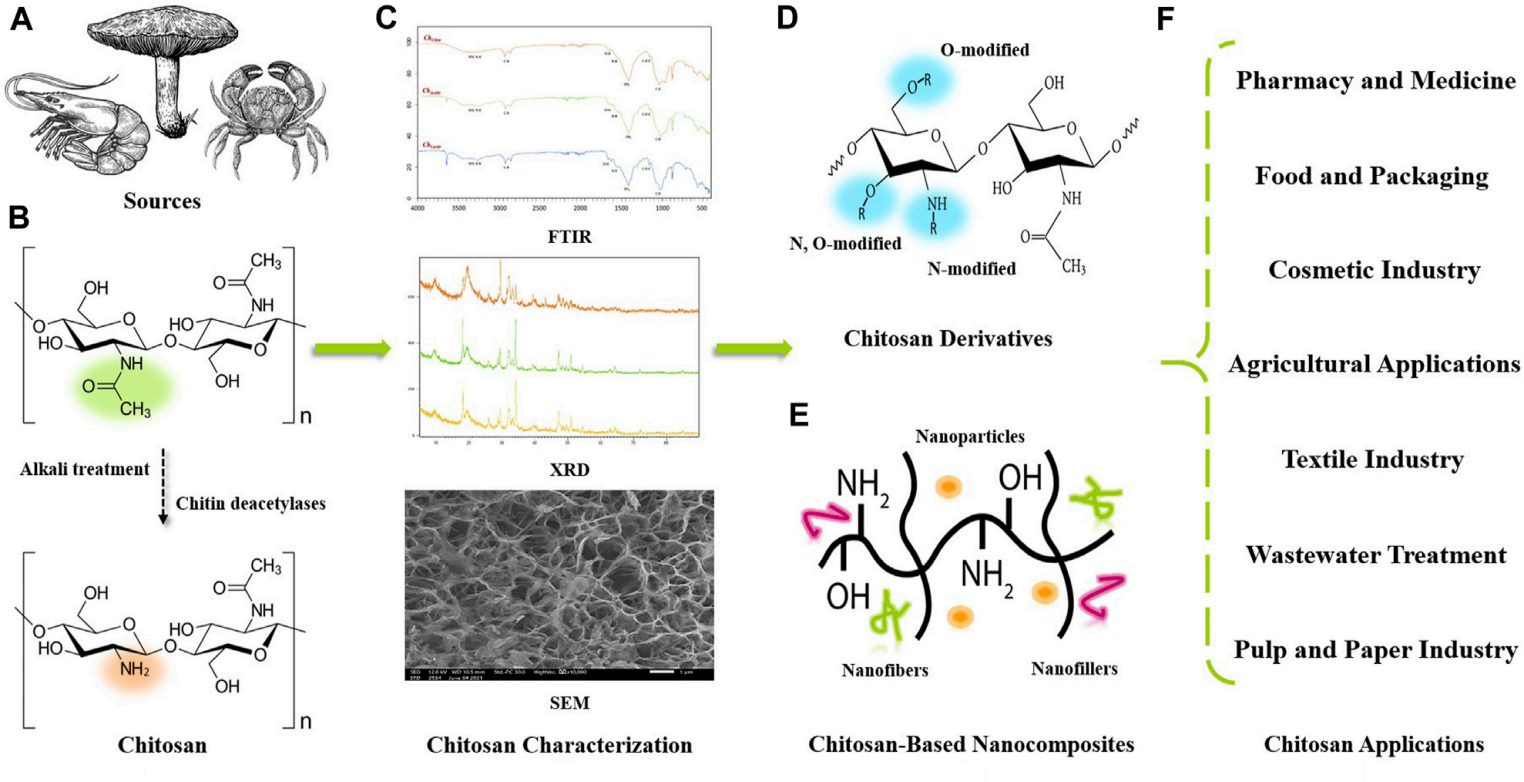
Schematic illustration from chitosan origin to potential applications in different emerging fields. **(A)** Various chitosan sources. **(B)** Conversion from chitin to chitosan by chemical or enzymatic deacetylation process. **(C)** Chitosan characterization by FTIR, XRD, and SEM. **(D)** N; O; or N, O-modified chitosan derivatives form. **(E)** Chitosan-based nanocomposites preparation. **(F)** Various chitosan engineering applications.

Today, the range of applications for biopolymers has broadened with the demand for innovative and environmentally friendly materials offering enhanced properties. Chitin, the parent compound of chitosan and the second most abundant biopolymer in nature after cellulose, is a linear polysaccharide composed of poly-*β*-[1,4]-N-acetyl-D-glucosamine units (Kou et al., 2022). This green polymer can be obtained from a variety of sources especially crustacean exoskeletons and its extraction requires several steps including demineralization, deproteinization, and decolorization (Jiménez-Gómez and Cecilia, 2020). The structure of completely acetylated chitin limits its applications due to its high thermochemical stability and its high insolubility in aqueous solutions and most organic solvents (Khajavian et al., 2022). However, chitosan is defined as the N-deacetylated form of chitin obtained by a chemical or enzymatic deacetylation process, and is also found naturally in the cell walls of certain fungi. It is a linear copolymer composed of repeated hydrophilic units (D-glucosamine units) and residual hydrophobic units (N-acetyl-D-glucosamine units) (Yarnpakdee et al., 2022). Molecular weight (Mw) and degree of deacetylation (DD) are the most characteristic features that influence the structure-function relationships of chitosan and its derivatives (Joseph et al., 2021). The solubility of chitosan is mainly linked to the amino groups along the polymer chain, which are fully protonated under acidic conditions, making chitosan highly soluble. This solubility generally increases with increasing degree of deacetylation and decreasing molecular weight (Mukhtar et al., 2021). Due to its cationic nature, chitosan has good biological activities such as bacteriostatic, antioxidant, antitumor, anti-inflammatory and antifungal activity (Aranaz et al., 2021; Ke et al., 2021). This natural bioactive polymer exhibits high biocompatibility, excellent biodegradability, exceptional functionality, cost-effectiveness, and non-toxicity, making it useful and powerful in various engineering applications. Figure 1 shows a schematic illustration from the origin of chitosan to its potential uses.

The presence of functional groups, amino (-NH_2_) and hydroxyl (-OH), in chitosan is a key attribute for its versatile chemical modification to improve its physicochemical, mechanical, and biological properties while maintaining its unique characteristics (Negm et al., 2020). Moreover, researchers have actively pursued the development of various chitosan derivatives to introduce innovative functions or properties to the material. The rapid strides in nanotechnology have notably intensified the focus on chitosan, positioning it as a highly sought-after matrix for nanocomposites. This heightened attention has not only bolstered the material’s efficiency but has also broadened its application spectrum across diverse fields. The synergy of chitosan and nanotechnology holds immense promise, unlocking new possibilities for advancements in materials science and technology (Yu et al., 2021). Chitosan, whether in its native state, derived or incorporated into nanocomposites, is an indispensable element in food packaging and preservation. This indispensability is attributed to its remarkable film-forming ability and outstanding antimicrobial properties. Chitosan’s inherent versatility enables it not only to improve the structural integrity of packaging materials, but also to act as an effective barrier to microbial activity, making a significant contribution to extending the shelf life of various food products (Hameed et al., 2022; Oladzadabbasabadi et al., 2022). Consequently, the application of chitosan in various forms underlines its crucial role in ensuring the safety and longevity of packaged foods. Chitosan and its derivatives, as well as chitosan-based nanocomposites, have attracted considerable scientific attention in the agricultural sector, mainly due to their notable impact on stimulating plant growth. This increased interest testifies to the versatility of chitosan’s applications in promoting agricultural sustainability and productivity (M. Zhang et al., 2022). The unique characteristics of chitosan, whether in its original form or in the form of modified derivatives and nanocomposites, have promoted its popularity for a variety of applications in different biomedical fields. These include its essential role as a diagnostic and therapeutic tool, as well as its incorporation into cosmeceuticals and cosmetics (Gao and Wu, 2022; Gupta et al., 2022). In the world of biopolymers, chitosan, in derived or nanocombined form, stands out for its relevance in a variety of absorption-related applications. These include its effectiveness in removing heavy metal ions and anionic dyes (Haripriyan et al., 2022). In summary, the versatility of chitosan allows it to be tailored to specific goals across a wide range of applications, making it a valuable material in many industries. This article aims to present an updated overview of chitosan production sources and extraction methods. The physicochemical and functional properties of the biopolymer were also emphasized. In addition, the review highlights methods for chitosan modification and the preparation of chitosan-based nanocomposites. This paper also gathers updated knowledge on the versatile applications of chitosan and its derivatives in diverse fields, such as the food industry, agriculture, the cosmetic industry, the pharmaceutical industry, medicine, and wastewater treatment.

## 2 Sources of chitosan production

Chitin is a polysaccharide naturally found in the exoskeletons of crustaceans, insects and mollusks, and in the cell walls of certain algae and fungi (Santos et al., 2020; Pellis et al., 2022). In addition, chitin is associated with other constituents such as minerals, proteins, lipids and pigments, whatever the initial extraction source. In general, the chitin content varies on average from 20% to 30% in crustacean exoskeletons (Kou et al., 2021), from 5% to 25% in insect cuticles (Zainol Abidin et al., 2020), and from 2% to 44% in fungi cell walls (Abo Elsoud and El Kady, 2019). The α-chitin, β-chitin and γ-chitin are the three allomorphic crystalline forms of chitin that differ in the unit size, number of chains, and degree of hydration. The α-microfibril is the most stable crystalline form of chitin and is found mainly in the exoskeleton of arthropods (Pellis et al., 2022). Shell waste from the seafood industry is a valuable source of raw materials and can be utilized to generate high value-added co-products and reduce environmental impacts, thus ensuring the sustainable development of the seafood industry. These economic and environmental advantages favor the utilization of crustacean sources, in particular crab and shrimp shells, for chitin and chitosan extraction, due to their availability as waste products and ease of extraction. Shrimp shells contain on average 30%–40% chitin, while crab shells contain 15%–30% (Pakizeh et al., 2021; Terkula Iber et al., 2022). Previous studies have shown that the source of the polymer and the extraction process have a direct impact on the physicochemical and biological properties of chitosan.

## 3 Chitosan extraction methods

### 3.1 Chemical extraction

**FIGURE 2:**
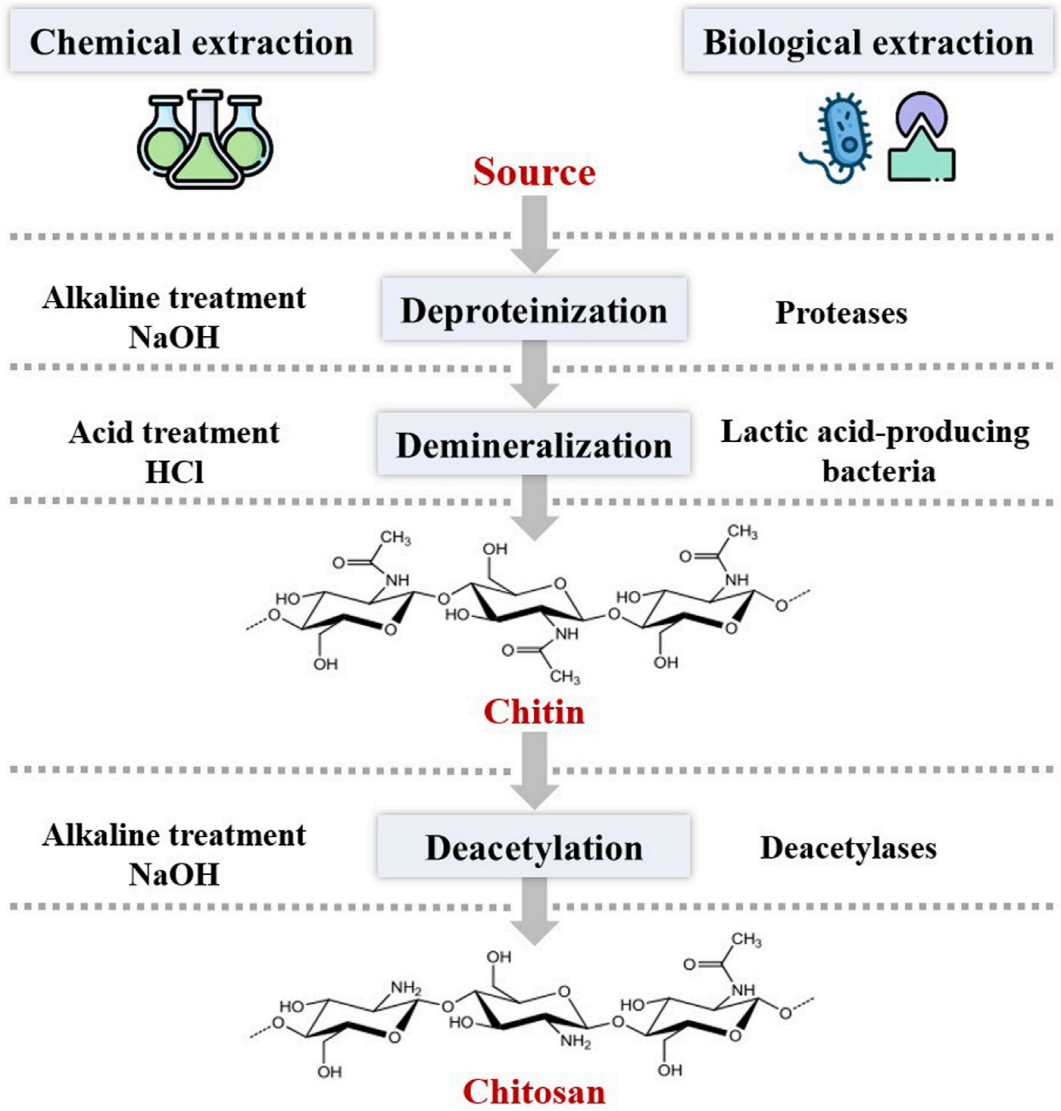
Chitin and chitosan obtained by chemical and biological methods.

Chitin is found mainly in association with minerals, proteins, glucans, pigments, and lipids. The abundance of these components varies according to the source and species of chitin. This variation requires a special extraction process that involves demineralization, deproteinization, and discoloration steps (Figure 2). The extraction of chitosan from crustacean sources is widely discussed in the literature. Demineralization of shellfish is a critical step due to the presence of minerals. This process occurs via the decomposition of calcium carbonate into calcium chloride, resulting in the release of carbon dioxide through acid treatment (Santos et al., 2020). According to the literature, sulfuric, hydrochloric, formic, acetic, oxalic, and nitric acids are generally used for their effectiveness in removing inorganic salts (Srinivasan et al., 2018). Efforts have been made to replace these mineral acids with more environmentally friendly organic acids. El-araby et al. (2022a) developed an ecological and economical demineralization step for chitosan extraction from shrimp shell waste using citric, acetic, and lactic acids. The results showed that during the demineralization phase, mineral acids can be replaced by organic acids for environmentally friendly extraction. The elimination of associated proteins is an important step in the chitin purification process. The deproteinization step consists in destroying the chemical bonds linking proteins and chitin by means of an alkaline treatment (Yadav et al., 2019). This step is usually done by strong bases, mostly with NaOH, at high temperatures and during a long incubation time (Pellis et al., 2022). Discoloration or bleaching is an optional step in the extraction process to remove pigments naturally present in chitin sources. This step requires the use of organic or inorganic solvents such as sodium hypochlorite, hydrogen peroxide, and acetone (El Knidri et al., 2018). Deacetylation is a final step in the extraction of chitosan and consists of the elimination of acetyl functional groups (-COCH_3_) from the chitin linear chain, with the release of amino groups (-NH_2_). The deacetylation process is generally carried out by heat treatment with a concentrated alkali, such as sodium hydroxide or potassium hydroxide (Kou et al., 2021). The proportion of acetylated versus deacetylated glucosamine units is important in determining the balance between hydrophilic and hydrophobic residues. Based on this, chitin with at least 75% deacetylation degree is called chitosan (Confederat et al., 2021).

### 3.2 Biological extraction

Microbial fermentation and enzymatic processing have been developed to overcome these problems and produce chitosan from crustacean by-products in a more environmentally friendly and cost-effective manner. The enzymatic extraction can be substituted during the deproteinization and the deacetylation steps to replace alkalis and high reaction temperatures with enzymes (Marzieh et al., 2019). Nevertheless, this method shares the similar demineralization process as chemical methods (Figure 2). Various proteases have been used for the enzymatic deproteinization of chitin, and these enzymes are generally extracted from fish viscera or microbes (Kou et al., 2021). In addition, various deacetylases derived from various biological sources such as bacteria, fungi, and certain insects have been employed for the enzymatic deacetylation step of chitin to obtain chitosan (Pellis et al., 2022). Extraction by microbial fermentation can be used in deproteinization, demineralization, and deacetylation steps, using proteases and lactic acid-producing bacteria (Doan et al., 2019). These fermentation processes for chitin extraction exploit the ability of bacteria to produce proteases such as *Pseudomonas aeruginosa*, *Pseudomonas maltophilia*, *Bacillus subtilis*, and *Serratia marcescens* (Navarrete-Bolaños et al., 2020).

### 3.3 Advantages and disadvantages of the two extraction methods

The chemical method produces chitosan with a high degree of deacetylation, medium to low molecular weight, and stronger biological properties. This method also involves a short treatment time and can therefore be easily applied on an industrial scale. This multi-step process, including the deacetylation step, implies the usage of strong bases and acids, which causes negative effects on the environment, diminishes the economic sustainability of the extraction process and often leads to a decrease in the quality of the resulting product. Furthermore, biological methods have significant limitations. The most important is the high cost of the enzymes involved in the enzymatic processing, which usually requires one or more different enzymes for deproteinization and deacetylation. Microbial fermentation indeed has the advantage of reducing the high cost of enzymes, but it generally requires specific microbial strains. Thus, unlike chemical extraction, biological extraction requires a long processing time (several days), which limits its use on an industrial scale. Despite the progress made to develop efficient and eco-friendly chitosan extraction methods, chemical extraction is the preferred method to date, due to the availability of chemicals and the possibility of industrial scale-up.

## 4 Physicochemical properties

### 4.1 Deacetylation degree

Chitosan is composed of three reactive functional groups, an amino group, primary hydroxyl group, and secondary hydroxyl group in each glycosidic unit. However, the -NH_2_ group is responsible for the cationic nature and physicochemical properties of chitosan thanks to its intramolecular and intermolecular hydrogen bonds (Bakshi et al., 2020). The physicochemical properties of chitosan depend on many factors, mainly the deacetylation degree (DD), the molecular weight (Mw), the solubility, and the degree of crystallinity. The distinctive feature of chitosan consists on its high DD and its low proportion of crystalline zones (Azmana et al., 2021; M. Zhang et al., 2022). The deacetylation degree of chitosan, the ratio of D-glucosamine (deacetylated unit) and N-acetyl-D-glucosamine (acetylated unit) structural units, increases proportionally with increasing alkali treatment time. As it is known, chitosan is a deacetylated form of chitin, with at least 75% deacetylation degree (Confederat et al., 2021). A high degree of deacetylation reflects a higher concentration of free -NH_2_ groups in the polymer chain and allows the molecule to exhibit enhanced biological activities and higher water solubility due to protonation of the amino functional group (Abd El-Hack et al., 2020). The degree of deacetylation of chitosan is one of the critical parameters influencing its physicochemical, mechanical, and biological properties and, therefore, its application spectrum. Table 1 summarizes sources, extraction methods, and physicochemical properties (yield, DD, solubility, and Mw) of chitosan obtained in various studies.

**TABLE 1:** Sources, extraction methods, and physicochemical properties of chitosan obtained in various studies.

Sources	Extraction methods	Yield (%)	Degree of deacetylation	Solubility	Molecular weight	References
Shrimp shells (*Litopenaeus vannamei*)	*Biological extraction* (Lactic bacteria and deacetylases)	74	78%	25%	71.31 kDa	Sixto-Berrocal et al. (2023)
*Eupolyphaga sinensis* walker	Chemical extraction (HCl and NaOH treatments)	5.48	96.57%	—	127.79 kDa	Jiang et al. (2023)
White shrimp (*Penaeus vannamei*)	Proteases and microwave heating on chitin deacetylation	17.54	90.75%	90.88%	67.88 kDa	Dong et al. (2023)
Beetles (*Pimelia payraudi latreille*)	*Acid-base extraction and discoloration*	39	90%	—	—	Amor et al. (2023)
Crab shells (*Gecarcinucidea* sp.)	Chemical isolation (HCl and NaOH treatments)	15.11	81.17%	65.94%	—	Wahab et al. (2023)
Shrimp shells (*Litopenaeus vannamei*)	Biological extraction (chitin deacetylase producing bacterial strain)	19.04	74.9%	71%	246.4 kDa	Rakshit et al. (2023)

### 4.2 Molecular weight

Besides the degree of deacetylation, the molecular weight (Mw) is another key factor that significantly affects the physicochemical properties of chitosan and, therefore, its biofunctionality and biological activities (Kou et al., 2021). Polymer viscosity is a parameter of considerable practical interest, as highly viscous solutions are difficult to handle, and it decreases, as the Mw of chitosan is reduced (Aranaz et al., 2021). Several studies have revealed that chitosan with a lower Mw and a higher DD generally exhibits higher bioactivities. In addition, previous studies have described different molecular weight threshold values for differentiating between high and low molecular weight chitosans. High Mw chitosans ranged from 190 kDa to 375 kDa, while low Mw chitosans ranged from 20 kDa to 190 kDa (Azmana et al., 2021). In general, the difference in molecular weight is related to the extraction process and the initial source of chitosan (Bakshi et al., 2020). Chitosan oligosaccharides (COS), degraded polymer products of polymer obtained by physical, enzymatic, or chemical hydrolysis, have gained attention in recent years owing to their lower Mw, higher DD, lower polymerization degree, low viscosity and greater water solubility. These physicochemical properties have significant beneficial effects and strong potential for biomedical, pharmacological, and industrial applications (Benchamas et al., 2021). Molecular weight has a major influence on the rheological properties of chitosan, which has a direct impact on the formulation of chitosan-based nanocomposites.

### 4.3 Solubility

The higher the DD of chitosan, the greater the degree of protonation of the -NH_2_ groups in the linear molecular chain, which facilitates its dissolution in acidic aqueous media, since its pKa value is around 6.5. This solubility is also influenced by the degree of deacetylation of chitosan. However, the higher the Mw of chitosan, the greater the number of intramolecular and intermolecular hydrogen bonds formed in its molecular chain, making it intertwined and therefore difficult to dissolve (Wang et al., 2020a; Aranaz et al., 2021). In addition, the polymer solubility depends on the position of acetyl groups along the linear chain, deacetylation procedure, and ionic strength. Various studies have shown that chitosan has great solubility when the degree of deacetylation exceeds 85% (M. Zhang et al., 2022). The low solubility of chitin in water and most organic solvents has limited its uses and applications. In contrast, the presence of -OH and -NH_2_ functional active groups in chitosan allows the formulation of a variety of derivatives that enhance its solubility, and therefore, increase the spectrum of applications in various fields. Carboxymethylation is an alternative method to improve the solubility of the biopolymer in aqueous solution (Jiménez-Gómez and Cecilia, 2020). Similarly, the quaternization of chitosan increased its solubility compared to unmodified chitosan (Wei et al., 2019). Chemical modification can both improve the physicochemical characteristics and biological properties of chitosan while retaining its unique properties and extend the range of chitosan derivatives applications.

## 5 Functional properties

### 5.1 Antibacterial activity

Chitosan is known as one of the most effective antibacterial substances because of its potential antibacterial properties. This biopolymer has been shown in previous studies to have antimicrobial activity against a wide range of Gram-positive bacteria (Savin et al., 2020; Valdez et al., 2022) and Gram-negative bacteria (Sudatta et al., 2020; Xing et al., 2021). The antibacterial properties of chitosan are influenced by numerous factors such as the molecular weight (Mw) of chitosan, the degree of deacetylation (DD), the polymer concentration, the initial source of chitosan, the pH value, and the storage temperature of the chitosan solution. Table 2 summarizes studies on chitosan’s antibacterial activity with respect to its DD and Mw. Likewise, it was found that the type of microorganism and the cell growth phase have a direct effect on the antibacterial potential of chitosan (Li and Zhuang, 2020; Aranaz et al., 2021). According to the literature, the higher the degree of deacetylation of chitosan, the stronger its antibacterial activity. Additionally, studies have shown that the antibacterial properties of chitosan solutions are inversely proportional to their molecular weight. This activity could increase with increasing molecular weight in an acidic medium and decrease in a neutral medium. This shows that the antimicrobial activity could be affected by the pH of its dissolution solution (Benhabiles et al., 2012; Chang et al., 2015). The main mechanism of action of chitosan’s antimicrobial activity is its interaction with the cell wall, cell membrane, and cytoplasmic constituents of bacteria (Abd El-Hack et al., 2020). The type of microorganism is known to have a significant influence on the antibacterial capacity of chitosan and its derivatives. The interaction of positively charged chitosan with the negatively charged microbial membrane of Gram-negative bacteria via electrostatic interactions could increase wall permeability by replacing divalent cations (Ca^2+^, Mg^2+^) from their binding sites and reducing the interaction between lipopolysaccharide molecules, thereby causing membrane rupture and cell lysis, leading to bacterial death. For Gram-positive bacteria, the absence of the outer membrane leads to a direct diffusion of chitosan into the bacterial cell wall (Duan et al., 2019; Wang et al., 2020a). This may explain the hypothesis that chitosan is more powerful against Gram-positive bacteria than Gram-negative bacteria.

**TABLE 2:** Studies on chitosan’s antibacterial, antifungal, and antioxidant activity with respect to its DD and Mw.

Functional properties	Physicochemical properties	Effects	References
Antibacterial activity	DD 85.61%	Complete inhibition of *Escherichia coli* and *Staphylococcus aureus* growth	Xing et al. (2021)
	Nanoparticles		
	DD 85%	Higher inhibitory potential towards *Staphylococcus aureus* compared to *Pseudomonas aeruginosa*	Savin et al. (2020)
	Mw 65.68 kDa		
	DD >85%	Bactericidal activity on *Bacillus cereus* growth and toxin formation	Valdez et al. (2022)
	DD 75%–85%	Antimicrobial potential against *Neisseria gonorrhoeae* growth	Alqahtani et al. (2020)
	Mw 50–190 kDa		
	DD 59.76%	High antibacterial activity against *Salmonella typhi*	Sudatta et al. (2020)
Antifungal activity	DD 80.86%	Total inhibition of *Aspergillus niger* mycelial growth	El-araby et al. (2022b)
	DD 95%	Complete inhibition of *Phytophthora infestans* mycelial growth and significant inhibition of spore germination rate	Huang et al. (2021)
	Mw 100 kDa		
	DD 75%–85%	Significant inhibition of *Fusarium sambucinum*, *Fusarium oxysporum*, and *Fusarium graminearum* mycelial growth	Mejdoub-Trabelsi et al. (2020)
	Mw 150 kDa		
	DD 93%	Inhibitory effects on spore germination and mycelial growth of *Aspergillus ochraceus*	Meng et al. (2020)
	Mw 100 kDa		
	DA 5.9%–6.8%	Fungistatic effect against *Penicillium citrinum* and *Penicillium mallochii*	Coutinho et al. (2020)
	Mw 132–245 kDa		
Antioxidant activity	DD 89%	Scavenging capacity of ABTS radical and DPPH radical	Savin et al. (2020)
	Mw 47.65 kDa		
	DD 92.4%	Higher DPPH radical scavenging activity and greater total reducing power capacity	Binh et al. (2021)
	Mw 65 kDa		
	DD 95%	Highest hydrogen peroxide, DPPH radical, and chelating ferrous ion (Fe2+) scavenging abilities	Chang et al. (2018)
	Mw 2.2 kDa		
	DD 78.60%	Best DPPH radical scavenging potential	Kusnadi et al. (2022)
	Mw 116.85 kDa		
	DD 80.43%	Significant DPPH free radical scavenging activity	Liyanage et al. (2022)

### 5.2 Antifungal activity

Various studies have reported that chitosan exhibits exceptional antifungal properties (Shih et al., 2019; Ashrafi et al., 2020) against a broad spectrum of molds and yeasts (Bernabé et al., 2020; Mejdoub-Trabelsi et al., 2020). The degree of deacetylation and the molecular weight are two parameters that play a critical role in controlling the antifungal properties of chitosan. Table 2 summarizes studies on chitosan’s antifungal activity with respect to its DD and Mw. In addition to its antifungal activity, low-molecular-weight chitosan is capable of penetrating the cell wall and surface, inhibiting DNA/RNA and protein synthesis (Kravanja et al., 2019). Similarly, the minimum inhibitory concentrations of chitosan against fungi were strongly correlated with the pH of the solvent and the type of fungus targeted (Garcia et al., 2018; Lopez-Moya et al., 2019). The antifungal activity of chitosan also depends on the concentration of the biopolymer. Studies have reported that concentrations of chitosan between 1% and 5% have been revealed to provide optimal antifungal activity Confederat et al., 2021). Chitosan is reported in the literature to have strong antifungal activity against chitosan-sensitive fungi, and the hypothesis predicts that positively charged chitosan can interact with negatively charged phospholipids in the fungal cell membrane, provoking membrane damage and entry into the cytoplasm (Verlee et al., 2017; Azmana et al., 2021). Chitosan-resistant fungi cell membrane constitutes a barrier to the polymer, which remains on the outer surface. In general, chitosan antifungal potential is considered to be fungistatic rather than fungicidal, and highly effective in inhibiting spore germination, radial growth, and germ tube elongation (Qin et al., 2020). The modification of chitosan is easily achievable thanks to the presence of -OH and -NH_2_ groups, which enhances the polymer’s antimicrobial properties and therefore its antimicrobial range.

### 5.3 Antioxidant activity

Chitosan and its derivatives have been shown to be powerful and potential antioxidants (Binh et al., 2021; Kusnadi et al., 2022). This antioxidant action appears to be highly correlated with the characteristics of the chitosan studied, and is closely related to the degree of deacetylation and molecular weight. Table 2 summarizes studies on chitosan’s antioxidant activity with respect to its DD and Mw. Chitosans with a lower molecular weight or a higher degree of deacetylation would have better antioxidant activity (Wang et al., 2020b). The NH_2_ and OH functional groups of this polymer are responsible for free radical scavenging and metal chelation (Chang et al., 2018; Aranaz et al., 2021). Chitosan antioxidant activity has been established by its strong hydrogen-donating capacity. Chitosan’s antioxidant action consists in protecting the target organism from oxidative stress-induced damage by interrupting the oxidation chain reaction (Muthu et al., 2021). Chitosan’s stability and reactivity are enhanced by chelation with another, more powerful antioxidant (Kadam et al., 2018; H. Zhang et al., 2018). For example, phenolic acids are perfect antioxidants, but they degrade rapidly in the body. Binding a slow-digesting compound such as chitosan inhibits its premature degradation by reducing its hydrophilicity (Carocho et al., 2018). It helps to stabilize phenolic compounds while maintaining biological chitosan properties. Chitosan chemical modification is possible due to its unique structure on which other active groups can be introduced to improve its solubility and bioactivity and generate new active functions (Qing et al., 2019; Sun et al., 2019).

## 6 Chitosan modification: Chitosan derivatives

**FIGURE 3:**
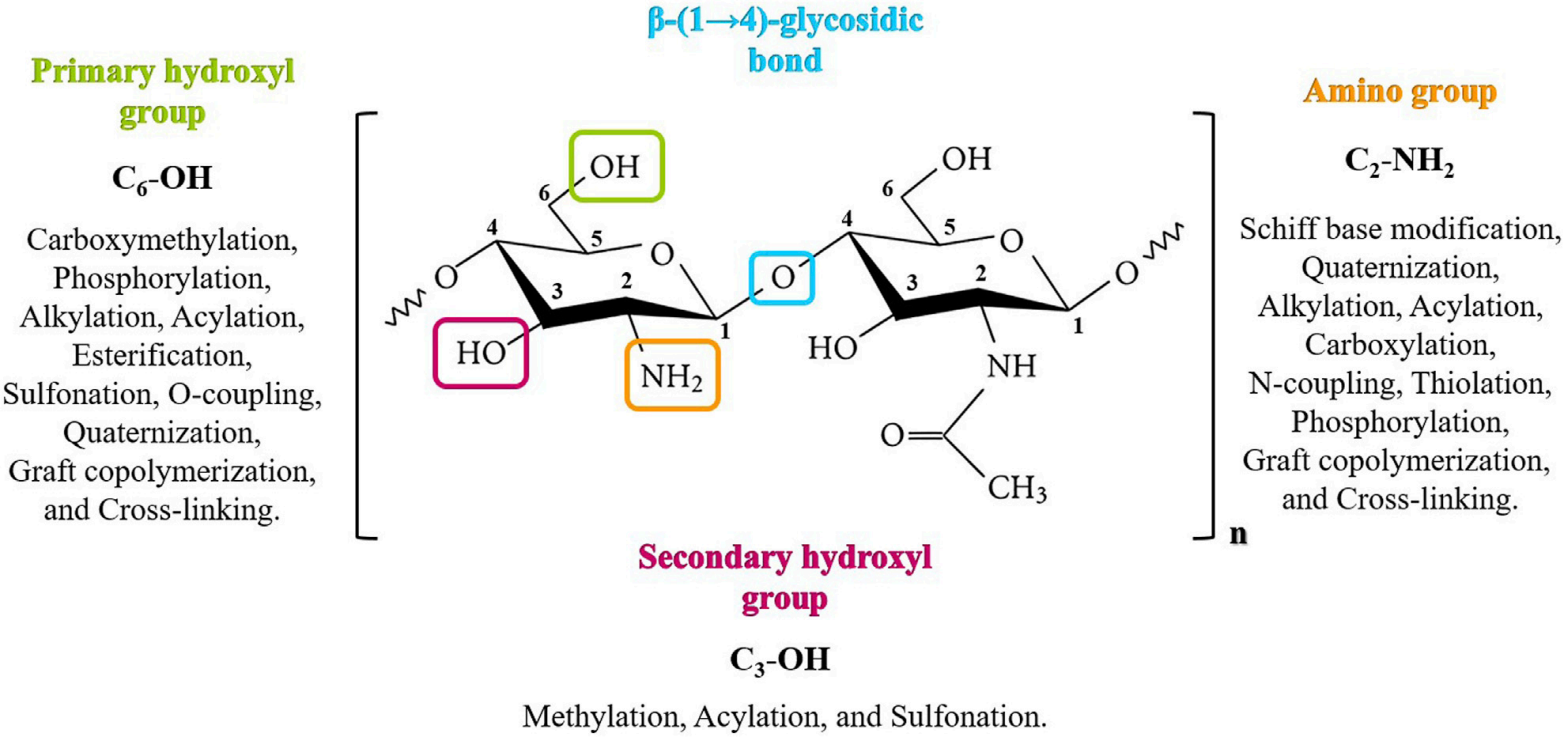
Structure and functional groups for the chemical modification of chitosan.

Chitosan is a versatile polymer that opens the possibility of various chemical modifications to produce a wide range of chitosan derivatives thanks to its hydroxyl and amino functional active groups. These modifications represent an effective way of improving the physicochemical characteristics of chitosan, while retaining its unique biological properties, leading to its increasingly widespread application in many fields (Jiménez-Gómez and Cecilia, 2020). The chemical structure of chitosan is characterized by NH_2_, primary OH, and secondary OH groups in positions C_2_, C_6_, and C_3_, respectively (Aranaz et al., 2021). The C_3_-OH group has a high resistance to the space site and is, therefore, relatively difficult to modify. In general, the order of reactivity of chitosan functional groups is as follows: C_2_-NH_2_> C_6_-OH> C_3_-OH (M. Zhang et al., 2022). Chemical modification of chitosan can occur at one of the active sites or equally at both sites to form N-, O-, or N, O-modified chitosan derivatives, and this can be achieved by a variety of chemical reactions (Figure 3). Chen et al. (2022) discuss the latest advances in chitosan chemical modification methods and review the uses of chitosan and its derivatives in several fields. The choice of modification depends on the desired properties and the intended use of the modified chitosan.

These modifications can be tailored to suit the properties required for specific applications in fields such as biomedicine, agriculture, food technology, wastewater treatment, and more. Here are some common chemical modifications of chitosan, for example, the alkylation reaction occurs by introducing an alkyl group onto C_2_-NH_2_ (N-alkylation) or onto C_6_-OH (O-alkylation) of the chitosan. The addition of an alkyl group weakens intermolecular hydrogen bonds, improving the solubility of the resulting molecule (Azmana et al., 2021). Phosphorylated chitosan is another derivative that can be obtained by reacting the polymer with phosphorus pentoxide in the presence of methane sulfonic acid at low temperature. The phosphorylated form of chitosan is recognized for its high water solubility, ability to chelate metals, and bactericidal properties (Negm et al., 2020). Quaternization of chitosan is a chemical modification achieved by incorporating quaternary ammonium groups or small-molecule quaternary ammonium salts into the C_6_-OH or C_2_-NH_2_ group. Quaternized chitosan exhibits greater solubility and antimicrobial properties than normal chitosan, favoring its use in biomedical fields (Wei et al., 2019). Thiolated chitosan is a derivative produced by the formation of a covalent bond between the thiol group (-SH) and the C_2_-NH_2_ group of chitosan, giving it high solubility in water compared with unmodified chitosan (Liu et al., 2021). In addition to the chemical processes described above to boost chitosan functional properties, cross-linking is a chemical process that can take place between molecules or inside the molecule and provides the polymer derivative greater stability through covalent bonding with the cross-linker (Saheed et al., 2021).

As research continues, the physical modification of chitosan is gaining importance to develop a new biomaterial with unique and distinctive physical characteristics to meet the needs of various applications. Physical modifications typically involve changes in the structure, morphology, or processing conditions of chitosan without introducing chemical alterations. This type of modification can be carried out by a variety of processes such as mechanical grinding, ultrasonic treatment, ionizing radiation, particle size reduction, blending, hydrogel formation, freeze-drying (Lyophilization), physical cross-linking, and heat treatment (Wang et al., 2020b). The biopolymer enzymatic modification is also possible for an ecological and less energy-consuming modification. The process involves the use of enzymes to introduce specific changes to the chitosan structure, often in a controlled and mild manner. Enzymes can selectively modify functional groups on chitosan, providing a route to tailor its biological properties for various applications (Bakshi et al., 2020). These enzymatic approaches offer a sustainable and biocompatible way of adapting chitosan to specific applications in fields such as biomedicine, food technology, and materials science, but they are still under development.

## 7 Chitosan-based nanocomposites

**FIGURE 4:**
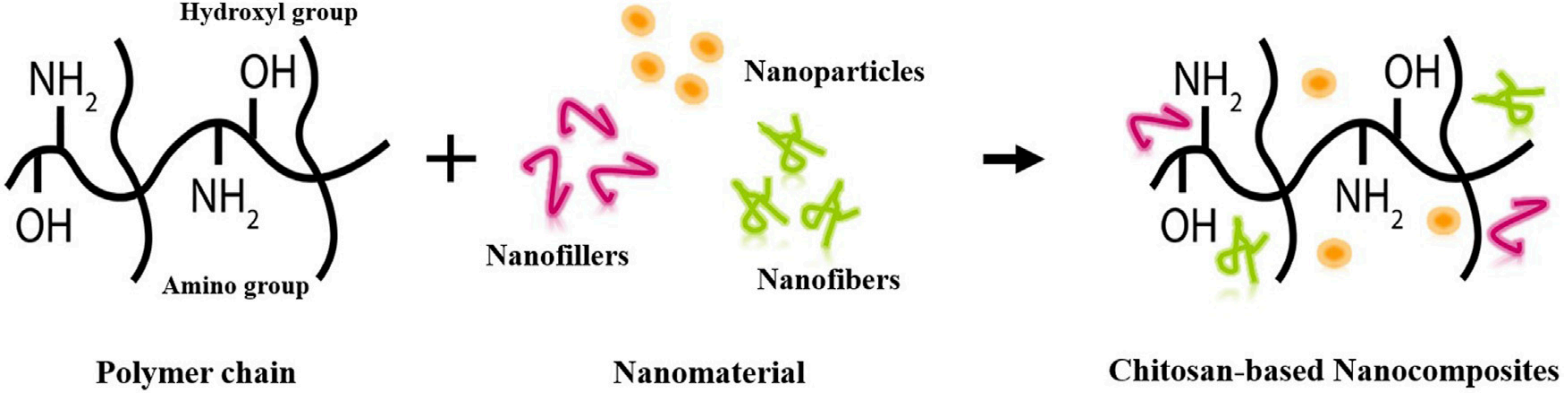
Chitosan-based nanocomposites preparation.

The presence of functional groups allows chitosan to be easily accessible to other biomaterials or active compounds, including biopolymers and metal ions, to form chitosan-based bionanocomposites. The availability and functionality make chitosan a promising candidate in the advanced field of nanotechnology. Chitosan-based nanocomposites are materials that combine chitosan with nanoscale additives such as nanoparticles, nanofillers and nanofibers (Figure 4) under controlled conditions to create a new material with enhanced properties (Azmana et al., 2021). These nanocomposites often exhibit improved mechanical strength, thermal stability, barrier properties and, other functionalities, making them suitable for a wide range of applications (Yu et al., 2021). Chitosan-based nanocomposites are synthesized by a variety of production processes, including solution casting, *in situ* synthesis, electrospinning, freeze-drying, layer-by-layer assembly, emulsion techniques, and the sol-gel method (Azmana et al., 2021). Each method offers unique advantages and allows for the tailoring of chitosan-based nanocomposites for various purposes. In fact, ongoing research is aimed at continuously improving production methods for chitosan-based nanocomposites, in order to increase their efficiency, properties, and applicability.

Chitosan-based nanocomposites offer a versatile platform for tailoring materials with enhanced properties for specific applications, ranging from medicine to packaging and beyond. The choice of nanoscale additive depends on the desired properties and the intended use of the nanocomposite. For example, chitosan-based nanocomposites obtained with cerium IV - zirconium IV oxide nanoparticles have shown remarkable improvements in morphological, structural, thermal, and mechanical properties. These nanocomposites, thanks to their developed properties, can be adapted to numerous applications such as the food packaging industry (Ahmed et al., 2021). Rodrigues et al. (2020) have developed chitosan-octadecylammonium and zinc oxide nanoparticles with improved mechanical, thermal, and antimicrobial properties, making them suitable for promising applications in active food packaging. In addition, chitosan-based nanocomposites are widely used as biological materials for wound and burn treatment, tissue engineering, and drug delivery systems (S. Ahmad et al., 2021). These nanocomposites, because of their small size and surface-to-volume ratio, are able to cross various biological barriers and deliver drugs to a specific site, making them promising to increase therapeutic efficacy in oral drug delivery (Khan and Alamry, 2021). Furthermore, chitosan derivatives via the cross-linking of polyethylene glycol diglycidyl ether using a microwave-assisted green method and its nanoparticles could potentially be used for biomedical applications (Ali et al., 2023). Nanocomposite materials can be also used in agricultural applications for controlled release of fertilizers and pesticides. Chitosan-based nanocomposites may also enhance plant growth and protect against pathogens (Yu et al., 2021). In addition, previous studies have revealed that chitosan-based nanocomposites are used for water purification and wastewater treatment. They can efficiently adsorb heavy metals, dyes, and other pollutants due to the high surface area and adsorption capacity of nanomaterials (Ahmad N. et al., 2020). The versatility of chitosan-based nanocomposites makes them suitable for a wide range of applications, and ongoing research continues to explore new possibilities and optimize their performance in different industries.

## 8 Application of chitosan, chitosan derivatives, and chitosan-based nanocomposites

**FIGURE 5:**
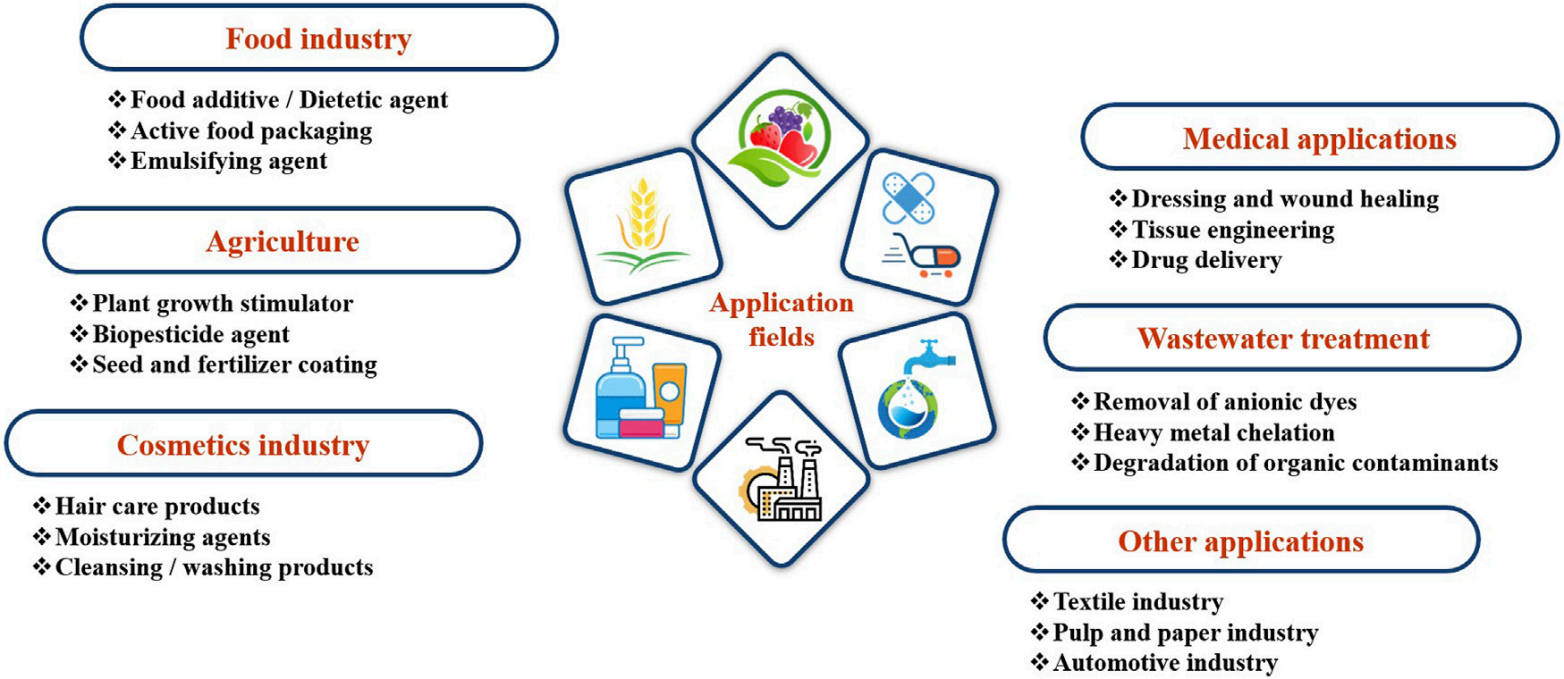
Applications fields of chitosan, chitosan derivatives, and chitosan-based nanocomposites.

Chitosan and its derivatives stand out as transformative materials with remarkable attributes, making them game-changing materials in a myriad of industries, including the food sector, agriculture, cosmetics, pharmaceuticals, medicine, and wastewater treatment (Figure 5). The availability, cost-effectiveness, non-toxicity, and versatile characteristics of chitosan and its derivatives make them indispensable materials in advancing sustainable practices and innovations across various fields. The broad spectrum of applications highlights the versatility and utility of chitosan, its derivatives, and nanocomposites in addressing challenges in a variety of sectors.

### 8.1 Food industry

#### 8.1.1 Food additives

Chitosan and its derivatives have a wide range of uses as food additives, thanks to their bioactive and cationic nature. In addition, chitosan is specifically biocompatible and biodegradable substance, which allows it to be broadly applied in the food industry (Morin-Crini et al., 2019b). Several studies have examined the safety and efficacy of chitosan and its suitability as a natural food additive. It has recently been certified as Generally Recognized As Safe (GRAS) by the United States Food and Drug Administration (USFDA) (Hu et al., 2019). Chitosan is widely used for its hypocholesterolemic property. Miyazawa et al. (2018) investigated the effects of chitosan from mushrooms on dietary obesity by providing mice with a chitosan-administered high-fat diet. The chitosan groups showed marked suppression of body weight gain. In addition, a clear reduction in low-density lipoprotein cholesterol and an increase in high-density lipoprotein cholesterol were observed, suggesting that the administered mushroom chitosan improved blood lipid metabolism. The mushroom chitosan showed an anti-obesity effect by inhibiting the digestion and absorption of lipid molecules. The use of chitosan in winemaking is widespread (Castro Marín et al., 2021). The potential use of biocompatible chitosan as a healthy additive or as an alternative to sulfur dioxide (a chemical additive) to prevent the oxidative degradation of white wine has been proven. It was found that the use of chitosan prevents the formation of the 1-hydroxyethyl radical. In the same report, a direct (dose-dependent) OH radical scavenging effect was revealed up to 98% at 2 g/L of chitosan (Castro Marín et al., 2019). Likewise, chitosan can be utilized in mimetic food as a fat substitute. A study by Rios et al. (2018) explored the capacity of a chitosan derivative as a fat alternative in cake formulations. The addition of 2 g of succinyl chitosan to 100 g of wheat flour increased the emulsifying capacity of the flour. The succinyl chitosan revealed a great potential for use as a partial fat substitute (up to 50%) in cake recipes. This chitosan derivative also reduced the rate of cake hardening during storage. The polymer has been tested for its ability to prolong the conservation period of catfish fillets and protect them from storage conditions (4°C). Vacuum tumbling with a chitosan solution stabilized the quality and color of refrigerated fillets by preventing the growth of aerobic bacteria (Bonilla et al., 2018). Carboxymethyl chitosan, an ampholic derivative of chitosan, is widely used as an effective food additive. Zhu et al. (2022) found that carboxymethyl chitosan improved storage stability and textural properties of the dough by limiting water migration and delaying protein deterioration during freezer storage. Today’s consumer is more attracted to natural food additives than synthetic ones. Chitosan is a natural non-toxic polymer. If mixed with food products, it will be safe for human’s body and will preserve the freshness and prolong the shelf life of perishable food products by preventing microbial spoilage and chemical deterioration (Hameed et al., 2022).

#### 8.1.2 Food packaging

Chitosan is an eco-friendly material for active food packaging owing to its natural availability, non-toxicity, renewable nature, inherent biodegradablity, and ease of modification. This biopolymer has excellent film-forming and antimicrobial properties that allow it to be considered as a suitable alternative source of food packaging materials to replace non-biodegradable and non-renewable polymers (Morin-Crini et al., 2019a; Y.-L. Zhang et al., 2021). Chitosan, as an active food packaging, can be applied, alone or in combination, in the form of a comestible thin film or an edible coating. Coating is a thin layer developed to cover a food product, delivered in liquid form by dipping the product in a chitosan solution or by spraying the solution onto the product (Cazón and Vázquez, 2019). The application of a chitosan film or coating shows promise for extending the storage period of highly perishable fruit and vegetables during post-harvest processing (Hameed et al., 2022; Oladzadabbasabadi et al., 2022). Chitosan’s capacity to scavenge free radicals as a natural antioxidant can be seen as an advantage for its potential use as a food packaging material (Wang et al., 2020b). According to the literature, chitosan is widely applied as a promising food packaging material to control the microbial growth of food, prevent oxidative deterioration of products, maintain the quality and nutritional characteristics during conservation, and also extend the shelf life of food products. Table 3 represents different applications of chitosan derivatives and chitosan-based nanocomposites as active food packaging materials. Chitosan, as an active and promising packaging for food preservation, can be applied in its native, modified or combined form. The main advantage of chitosan is that it can undergo a wide range of chemical and physical modifications to improve its functionality (Inanli et al., 2020). Chitosan nanocomposites have been shown to improve the physical properties of the biopolymer, making it useful in various applications such as the food packaging industry.

**TABLE 3:** Applications of chitosan and its derivatives as active food packaging materials.

Packaging (Film/Coating)	Food	Main findings	References
Chitosan and chitosan nanoparticles coating	Banana fruit	Reduction of chilling injury and weight loss by increasing firmness, total antioxidant activity and total phenolic content	Elbagoury et al. (2022)
Chitosan film	Beef meat	Improvement of the microbiological quality and extension of shelf life during storage	Duran and Kahve (2020)
Chitosan film incorporated with citric acid	Matured cheese	Lower weight loss and antimicrobial activity against aerobic mesophilic bacteria	Ressutte et al. (2022)
Chitosan-olive oil coating	Fresh Figs	Delay of fungal rot and post-harvest ripening indicators	Vieira et al. (2021)
Chitosan nanoparticles composite films	Fish fillets	Extending the storage period of aquatic products by avoiding lipid oxidation and proteolysis	Zhao et al. (2022)
Chitosan-Ruta graveolens essential oil coatings	Tomatoes	Preservation of physico-chemical properties and delay or inhibition of the development of microbial spoilage	Peralta-Ruiz et al. (2020)
Chitosan coating and lauric arginate	Chicken drumsticks	Improvement of sensory scores, oxidative stability and antimicrobial quality of the frozen stored product	Abdel-Naeem et al. (2021)
Chitosan derivatives and polyvinyl alcohol films	Mangoes and papayas	Delayed senescence of the fruits, and extension of their shelf life	Pan et al. (2022)
Carboxymethyl chitosan-based coatings	Mushrooms	Improvement of the overall quality of mushrooms by reducing the total viable count, decreasing respiratory rate and weight loss, and inhibiting mushroom browning	Li F et al. (2022)
Chitosan coating	Strawberry fruit	Reduction of the microbial spoilage load, stabilization of the initial pigmentation and preservation of the cellular structures	El-araby et al. (2022b)
Chitosan coating	Vacuum-packaged beef	Reduction of lipid oxidation and inhibition of lactic bacteria growth	Gedarawatte et al. (2021)
Chitosan coating with green tea aqueous extract	Pork chops	Improvement of the physicochemical characteristics (pH, color, and oxidation of lipids) and microbiological properties of samples during conservation	Montaño-Sánchez et al. (2020)
Chitosan-propolis extract coating	Crayfish	Control of chemical indices and bacteria growth and also an extension of fish shelf life	Çoban (2021)

A study by El-araby et al. (2022b) evaluated post-harvest treatment of strawberries (*Fragaria x ananasa*) with chitosan solution as a preservative coating. The results showed that the coating reduced post-harvest losses and prolonged the storage period by 7–8 days by minimizing microbial spoilage load, maintaining anthocyanin pigments, and retaining fruit firmness during storage. Batista Silva et al. (2018) found that chitosan coating improved the shelf life and stabilized the overall quality of guava (*Psidium guajava L.*) fruits after harvest by enhancing antioxidant processes and retarding ripening while stored at room temperature. Chitosan coating has been shown to reduce fresh weight loss, preserve fruit skin firmness and color, and delay chlorophyll degradation without significant effect on titratable acidity. Chitosan films prepared by adding peanut skin and pink pepper residue extracts were studied as active packaging for chicken products. Treatment with active films guarantees the oxidative stability and quality of chicken products, thanks to antioxidant and antimicrobial performances (Serrano-León et al., 2018). A study by Gedarawatte et al. (2021) developed spray coatings of chitosan and gelatin to prolong the shelf life of vacuum-packaged beef. The edible chitosan coating significantly reduced lipid oxidation and prevented lactic acid bacteria growth when compared to uncoated and gelatin-coated beef samples. This study shows that chitosan coating in spray form is easily adaptable to industrial environments as an antimicrobial and antioxidant application prior to the meat vacuum packaging process. Y.-L. Zhang et al. (2021) evaluated the effect of an edible coating of carboxymethyl chitosan and gelatin on a variety of sweet cherry cultivars during post-harvest processing. The coating maintained the nutritional quality and properties of the sweet cherry varieties. The developed preservation method reduced weight loss and maintained initial peel color, stem freshness, firmness of the fruit, ascorbic acid content, titratable acidity, total phenolic content, and total anthocyanin concentration.

### 8.2 Agriculture

The world’s growing population, shrinking arable land, and the development of plant diseases are major challenges facing the agricultural sector, resulting in considerable economic losses. However, the continued use of fertilizers, pesticides, fungicides, and large amounts of chemical nutrients has an impact on the sustainable development of the agricultural economy and public health security. Hence the importance of developing modern agriculture that takes these challenges into account and that must be adapted to climate change. Chitosan and its derivatives have been largely proposed as environmentally friendly alternative sources to the application of agrochemicals. The bioactivities of chitosan such as antifungal activity, crop yield improvement, induction of plant defense system, and promotion of plant growth play a crucial role in its application in agriculture (Morin-Crini et al., 2019b). Chitosan, as a biostimulant, has the ability to act inside and outside the plants and apply physical or physicochemical effects on them. It is broadly recognized for its physiological effects on nutritional efficiency, and on the response to abiotic stress (Bhupenchandra et al., 2020). Several studies have indicated that chitosans induce the activities of genes responsible for various events in plant life processes, such as systemic acquired resistance, photosynthesis, plant defense system, hormone metabolism, and alteration of protein metabolism, resulting in increased storage protein content (Landi et al., 2017; Xoca-Orozco et al., 2017). The advantageous functions of chitosan are mainly related to the tolerance of plants to abiotic stress factors such as salinity, high temperatures and drought, as well as to the enhancement of their photosynthetic performance (Shahrajabian et al., 2021). Chitosan’s hydrophilic nature reduces transpiration rate and promotes water absorption. Rahman et al. (2018) revealed that foliar application of chitosan to strawberries improved plant growth and fruit yield. A significant increase in fruit weight and higher levels of anthocyanins, carotenoids, flavonoids, and phenolic compounds were observed when plants were sprayed with a chitosan solution compared to the untreated control. Another study reported that the biomass and number of flowers of mycorrhizal tomato plants were improved by foliar spraying with chitosan (El Amerany et al., 2020).

Saharan et al. (2016) report that Cu-chitosan nanoparticles, through α-amylase and protease activity, improve the growth of corn seedlings by exploiting reserved feed, mainly starch and protein. A study by Jogaiah et al. (2020) revealed that the application of chitosan to cucumber seeds had a positive impact on disease protection and improved plant development. The results show that chitosan increased phytohormone regulation and synthesized defense enzymes, inducing resistance to mildew disease, and enhancing cucumber plant development. In addition, chitosan-based biodegradable and biocompatible nanomaterials are employed in soil conditioners, seed coatings, and foliar treatments to promote plant growth and protect plants against fungi, bacteria and viruses, offering a novel and promising material for durable crop protection (Shahrajabian et al., 2021). Vanti et al. (2020) synthesized copper nanoparticles coupled with chitosan and evaluated their benefits on chili, cowpea, and tomato crops. The prepared nanoparticles had beneficial effects as fungicide and growth promoter and explored their possible application as a safe alternative to conventional pesticides to avoid hazardous effects on the environment. Apart from the direct effects of chitosan, chitosan nanoparticles have synergistic effects with plant-friendly metals such as Zn, Cu, Ag and Ni and can enhance their fungicidal and disinfectant properties. Table 4 represents other uses of chitosan derivatives and chitosan-based nanocomposites in agriculture. A study by Choudhary et al. (2019) showed that Zn-chitosan nanoparticles presented strong antifungal and growth-promoting activities in corn seedlings. Zn-chitosan nanoparticles have been shown to combat disease by boosting plant immunity through increased antioxidant and defense enzymes, regulation of reactive oxygen species and increased lignin uptake.

**TABLE 4:** Applications of chitosan derivatives and chitosan-based nanocomposites in agricultural sector.

Systems	Plants	Main findings	References
Potassium-chitosan nanoparticles	Maize	Improved soil physical properties by increasing porosity, water conductivity, and friability, which promoted root growth. Significant increase in fresh and dry biomass accumulation	Kubavat et al. (2020)
Chitosan-based nanocomposites loaded with antioxidants	Tomato	Linear inhibition of fungal pathogen growth in seedlings. Promising pesticides for seedling growth	Elsherbiny et al. (2022)
Chitosan-salicylic acid nanocomposites	Grapes	Enhanced physiological, biochemical, and elemental nutrient balance characteristics. Excellent biostimulant for the improvement of plant yield under salinity stress	Aazami et al. (2023)
Chitosan nanofertilizer comprising of copper and salicylic acid	Maize	Improved nutrient remobilization in growing cobs and increased source activity in developing plants. Foliar application increased antioxidant enzyme activities and increased chlorophyll content in leaves	Sharma et al. (2020a)
N, P, and K-chitosan nanoparticles	Potato	Increased photosynthetic pigments and macronutrients in leaves and tubers. Significant acceleration of plant development and productivity	Elshamy et al. (2019)
Zn-chitosan nanoparticles	Wheat	Improved stress resistance and antioxidant status of plants. Regulation of the starch biosynthesis process to increase source activity and sink strength	Kumar et al. (2021)
Chitosan and Moringa oleifera iron oxide nanoparticles	Corn	Positive effect on plants germination and growth without any toxic impact. Increased root and stem length	Tovar et al. (2020)
Chitosan based NPK-nanofertilizers	Cucumber	A significant increase in leaf area and yield characteristics. Improved efficiency of maximum apparent recovery of N, P, and K	Modi et al. (2021)
Chitosan nanoparticles	Rice	Improvement of the yield and biological properties of the plant. Excellent growth stimulator without any toxic effect	Divya et al. (2021)
Chitosan based NPK-nanofertilizers	Coffee	Improved nutrient uptake in the leaves, photosynthesis process, and plants growth. Increased leaf number, plant height, and leaf area	Ha et al. (2019)

### 8.3 Cosmetic industry

These days, bio ingredients are attracting a lot of interest in the cosmetics sector to overcome the undesirable effects of synthetic active ingredients, such as skin irritation, itching, photoallergy, and phototoxicity. Researchers are therefore increasingly interested in chitosan and its derivatives for the preparation of cosmetic products, due to its natural abundance, ease of extraction and excellent cosmetic qualities. Chitosan is the only natural cationic polymer whose bacteriostatic, fungistatic, moisturizing, and film-forming properties favor its use in cosmetic formulations (Jiménez-Gómez and Cecilia, 2020; Aranaz et al., 2021) and make it attractive in skin, nail, hair, and oral care applications (Morin-Crini et al., 2019a; Azmana et al., 2021). Chitosan is used in hair treatment products including shampoos, hairsprays and dyes to promote and boost the hair’s softness and mechanical strength, to eliminate oils and sebum due to its hydrophobic nature, and to maintain humidity and styling by reducing static electricity in the hair (Abd El-Hack et al., 2020). Thanks to its film-forming qualities, chitosan has the capacity to interact with hair keratin, creating a transparent elastic film on hair fibers, increasing smoothness and force and preventing damage (Wang et al., 2020b). One of the essential benefits of chitosan in the cosmetic field is its use in moisturizing agents to keep the skin well hydrated and nourished. It softens the epidermis and prevents damage caused by external environmental conditions and cleansers. High-molecular-weight chitosan has film-forming characteristics that can help reduce skin water loss, increase elasticity and improve skin smoothness (Casadidio et al., 2019; Gupta et al., 2022).

Petrick et al. (2020) synthetized a chitosan/TiO_2_ nanocomposite as a multifunctional sunscreen for moderate UV protection with an ability to inhibit the activity of bacteria living on the skin surface up to 99.7% in 2 h. The addition of chitosan to lip care products makes lips softer and protects them from drying out. It is also used as an active ingredient to promote long-term adhesion of lipstick colors (Bakshi et al., 2020). Due to their cationic nature, chitosan and its derivatives are suitable for inclusion in cleansing products such as cleansing milks, face toners, peels, soaps and shower products, by exploiting the ionic attraction between their cationic charge and the anionic nature of the skin surface (Abd El-Hack et al., 2020). Chitosan and its derivatives are widely applied in the treatment of oral problems, through the creation of dental gels, toothpastes, and mouthwashes (Gupta et al., 2022). A toothpaste developed with biosurfactants and chitosan had a positive effect on the inhibition of biofilm formed by *Streptococcus mutans* compared to the commercial toothpaste tested. The addition of chitosan and its derivatives has also been shown to improve the inhibition of dental biofilm (Resende et al., 2019). The deodorizing effect and antimicrobial characteristics of chitosan enable it to be used as an active ingredient in deodorizers, as it inhibits the activity of enzyme-producing bacteria (Bakshi et al., 2020). Table 5 represents other applications of chitosan derivatives and chitosan-based nanocomposites in the cosmetic industry. The cosmetics sector has developed very rapidly in recent times, and products based on chitosan alone or combined with other components are already available on the market.

**TABLE 5:** Applications of chitosan derivatives and chitosan-based nanocomposites in cosmetics.

Systems	Main findings	References
Carboxymethyl chitosan	A remarkable moisturizing effect due to the polymer’s ability to form a hydrated layer on the skin’s surface, preventing water loss	Chaiwong et al. (2020)
Chitosan-lemongrass essential oil films	Application as antioxidant and antimicrobial care masks with high flexibility and permeability, without cytotoxic risks	Gaspar et al. (2022)
Methylcellulose-chitosan smart gels	Heat protection with chitosan that acts like a film on the hair, protecting it from heat damage. Applied like an ultra-hold hairspray on hair, it fixes the style for hours	Hartson et al. (2022)
Carboxymethyl chitosan	Safety and efficacy of injectable soft tissue devices for the intradermal treatment of age-related skin defects. Significant improvement in skin hydration, firmness, and elasticity	Philippart et al. (2021)
Thymoquinone-loaded chitosan nanoparticles	Superior antimicrobial activity over time and a natural, effective, and lasting preservative effect in cosmetic products	Mondéjar-López et al. (2022)
Pomegranate juice-loaded chitosan derivative nanoparticles	Improved antioxidant and antimicrobial properties of skin emulsions with good storage stability. Higher sun protection factor	Bikiaris et al. (2020)
Carboxymethyl chitosan-mangosteen extract	Good moisturizing power for the skin and good deodorizing property against trans-2-nonenal odor, with antioxidant and antibacterial properties	Chaiwong et al. (2022)
Chitosan nanoparticles	A significant anti-sebum characteristic on the T-zone of the face after 4 weeks, without significant disruption of the skin barrier	Theerawattanawit et al. (2022)
N-[(2-hydroxy-3-trimethyl-ammonium)-propyl] chitosan chloride	Excellent antibacterial and mechanical properties, with desirable cell attachment and proliferation	Khalaji et al. (2021)
Chitosan/vitamin C complex	Enhanced antioxidant, moisturizing, antibacterial, and film-forming properties. High moisture retention, hygroscopicity, ability to scavenge hydroxyl radicals, and high stability	Liping et al. (2020)

### 8.4 Pharmaceutical and medical applications

#### 8.4.1 Wound dressing and healing

Chitosan has the ability to improve the wound-healing process and prevent bacterial infections due to its hemostatic, antimicrobial, anti-inflammatory, film-forming, and analgesic properties, making it useful as an active material for dressings (Abd El-Hack et al., 2020; M. Zhang et al., 2022). It contributes effectively to cell growth due to its positive surface charge, which has led to thrombosis and blood coagulation (Wang et al., 2020a). Chitosan-based bionanocomposites are utilized as wound healing constituents by enhancing the epithelialization process and collagen deposition on the dermal layer of the skin (Azmana et al., 2021). Chitosan-loaded cyclodextrin hydrogels decreased blood leakage and the duration of hemostasis compared with commercial hydrogels. The results show that artificial hydrogels hold promise as a physiologically safe means of mitigating blood loss in tissue injury situations (Leonhardt et al., 2019). The chitosan microneedle patch has proved to be very useful for wound healing, promoting inhibition of inflammation, collagen synthesis, and tissue renewal during wound healing (Chi et al., 2020). The chitosan nanosilver-based dressing had a greater and quicker healing effect than intradermal injection of mesenchymal stem cells. In the same report, the group receiving the developed dressing showed a significant increase in epidermal thickness, collagen density and nuclear antigen immunoreactivity of proliferating cells (Ghannam et al., 2018). Sulfated chitosan-type I collagen hydrogel accelerated the healing of chronic diabetic wounds by enhancing the functions of macrophages into fibroblasts, resulting in enhanced collagen and extracellular matrix formation in the wound tissue (Shen et al., 2020). Polyvinyl chitosan nanofibers were associated with carboxymethyl chitosan nanoparticles encapsulated with an antibacterial peptide. These nano-system revealed antibacterial properties and stimulated the healing of mouse tissue (Zou et al., 2020). Micro-channeled alkylated chitosan sponges have shown promising clinical translation potential to treat non-compressible lethal hemorrhages and facilitate wound healing. These hemostatic chitosan sponges demonstrated water and blood absorption capacity, and provided higher coagulant and hemostatic potencies in liver perforation wound models of lethally normal and heparinized rats and pigs, compared with treatments used in the clinic (Du et al., 2021). Chitosan functionalized via coupling of chitosan -NH_2_ groups with 2,4,6-trimethoxybenzaldehyde could be a promising candidate for wound dressing products and cutaneous cancer treatment (Tamer et al., 2023).

#### 8.4.2 Tissue engineering

Chitosan-based bioactive materials have emerged as promising candidates for tissue engineering applications, as they degrade progressively as new tissue is formed, avoiding inflammatory reactions and toxic degradation (Bakshi et al., 2020; Islam et al., 2020). Chitosan-grafted polymethyl methacrylate and modified hydroxyapatite have been successfully developed and applied for bone tissue engineering. The biocomposite exhibited good mechanical strength and long-term stability after soaking, and could be utilized as a scaffold for bone cell growth and drug delivery during bone repair (Tithito et al., 2019). Composite scaffolds of gelatin, chitosan, polyvinyl alcohol, and nano-hydroxyapatite showed positive effects on osteogenic differentiation and were capable of mimicking the structure and function of natural bone. The composite scaffolds effectively promoted cell proliferation and adhesion, making them a promising biomimetic scaffold for bone tissue engineering (Ma et al., 2021). The synthetized cellulose nanofiber-filled chitosan hydrogels have been effective in repairing and regenerating the annulus fibrosus tissue of the intervertebral disc. Nanocomposite material is suitable for use as an implant in annulus fibrosus tissue defects to repair intervertebral disc biomechanics and provide retaining plates against disc nucleus protrusion, while supporting intervertebral disc regeneration and approximating the functionality of a healthy disc (Doench et al., 2019). Pore scaffolds formulated with chitosan, gelatin, and silk proteins exhibited good chondrocyte cell viability and promoted rapid cartilage tissue regeneration in defective rabbit knee articular cartilage (Haghighi and Shamloo, 2021). Chitosan-vitamin C-lactic acid membranes have been used in skin tissue engineering. The prepared porous chitosan composite membranes provided an optimal environment for the attachment, growth, and spreading of skin cells (NIH-3T3 fibroblasts) compared to non-porous membranes (Madni et al., 2019). Composite scaffolds containing silk fibroin, carboxymethyl chitosan, strontium-substituted hydroxyapatite and cellulose nanocrystals proved effective in enhancing osteoblast adhesion and proliferation. The experimental results suggested the applicability of the synthesized scaffolds for bone repair (X. Zhang et al., 2019).

#### 8.4.3 Drug delivery

Biocompatibility, biodegradability, long-term stability, mucoadhesive capacity, non-toxicity, cationic nature, and the presence of amino and hydroxyl groups are the most critical characteristics that make chitosan an active and promising polysaccharide for various drug delivery (Barman et al., 2020; Hameed et al., 2022). Advances in drug delivery systems are attracting growing interest from researchers, and point to the need to develop innovative and improved materials using chitosan-based nanocomposites to enhance drug delivery efficiency. In general, microspheres, tablets, gels, microcapsules, and films with sustained release can be prepared by combining the polymer with drugs by dissolution, coating or adsorption (Wang et al., 2020b). Furthermore, the introduction of new entities into the polymer linear chain enhances other characteristics of chitosan, boosting its suitability for pharmaceutical purposes. For example, O-carboxymethyl and N-trimethyl chitosan are the most promising chitosan derivatives and have excellent potential for drug delivery system (Abd El-Hack et al., 2020). A drug delivery system is a technical device that combines medicine, engineering, and pharmacy to deliver the precise dose of drug to the right place at the correct time, increasing the drug’s bioavailability and therapeutic efficacy, and minimizing adverse effects (Ewart et al., 2019; Wang et al., 2020a). Table 6 represents chitosan-based nanocomposites in different drug delivery systems. Chitosan-based devices are used for the delivery of proteins and peptides, growth promoters, anti-inflammatory agents, antibiotics, and vaccines, also in gene therapy and bioimaging applications (Bakshi et al., 2020).

**TABLE 6:** Chitosan-based nanocomposites in different drug delivery systems.

Chitosan-based nanocomposites	Loaded drugs	Routes of administration	Main findings	References
N-trimethyl chitosan coated nanocomplexes	Gemcitabine	Oral delivery	Enhanced drug bioavailability, improved its therapeutic effect, and inhibited tumor growth	Chen et al. (2021)
Chitosan nanoparticles	Hesperidin	Nasal delivery	Improved cellular absorption and reduced cytokine storm syndrome in the lungs	Jin et al. (2021)
N-Trimethyl chitosan nanoparticles	Flurbiprofen	Ocular delivery	Delayed drug liberation and enhanced its therapeutic efficacy	Shinde et al. (2019)
Chitosan nanoparticles	Metformin	Oral delivery	Increased systemic delivery and therapeutic efficacy in polycystic kidneys	(J. Wang et al., 2021)
Carboxymethyl chitosan nanoparticles	Basic fibroblast growth factor	Transdermal delivery	Improved dosing efficiency and prevented the drug from remaining on the skin surface	Xie et al. (2022)
Chitosan nanoparticles loaded nanofiber	Benzydamine	Vaginal delivery	Increased mucoadhesion and provided higher permeation through the vaginal tissue	Tuğcu-Demiröz et al. (2021)
Chitosan hydrogel	Ibuprofen	Nasal delivery	Improved drug solubility and accelerated transport through nasal epithelial cells	Gholizadeh et al. (2019)
N-trimethyl chitosan coated nanoparticles	Vitexin	Oral delivery	Promoted drug absorption and improved its antioxidant activity	Li S et al. (2022)
Glycol-chitosan oxidize hyaluronic acid hydrogel film	Levofloxacin	Ocular delivery	Showed progressive drug release and significantly reduced various inflammatory cytokines	Bao et al. (2021)
Chitosan nanoparticles	Acyclovir	Vaginal delivery	Enhanced cellular absorption in the vaginal mucosa and prolonged drug liberation	Deshkar et al. (2021)
Thiolated chitosan microneedle patch	Tacrolimus	Transdermal delivery	Enhanced availability and sustained liberation over a longer period. Better penetration into the dermis, without rupture	Ahmad et al. (2020b)
N-Trimethyl chitosan nanoparticles	Metronidazole	Periodontal delivery	Enhanced antibacterial activity against periodontal infections	Garg and Tirgar (2022)
Chitosan-ethyl cellulose microspheres	Domperidone	Nasal delivery	Improved the bioavailability by avoiding its first-pass metabolism and regulated drug concentration in the blood	Zafar et al. (2021)

### 8.5 Wastewater treatment

The daily discharge of industrial wastewater into landfills results in significant water and environmental contamination. Physico-chemical treatments can be used to eliminate heavy metals and toxic compounds from wastewater. However, these methods are harmful to the environment due to the use of synthetic chemicals. The presence of active functional groups (-NH_2_ and -OH) has allowed chitosan and its derivatives to gain more popularity than other polysaccharides as an effective adsorbent for water purification. Chitosan-based bionanocomposites have been reported to be very useful as safe and environmentally friendly alternatives for the chelation of heavy metals and dyes in industrial wastewater. Cationization of -NH_2_ groups leads to absorption of anionic dyes through electrostatic attraction in acidic conditions (Thomas et al., 2019; Azmana et al., 2021). Microfluidically generated chitosan microspheres significantly eliminated Cu and other toxic heavy metal ions from the polluted water. The excellent adsorption capacity, biodegradable properties, and low extraction cost make chitosan microspheres a promising material in industrial wastewater treatment applications (B. Wang et al., 2019). Sessarego et al. (2019) synthesized a phosphonium cross-linked chitosan to eliminate Cr (VI) from wastewater by adsorption. The research indicated that phosphonium functionality is provided to the chitosan by tetrakis (hydroxymethyl) phosphate through an easy-on-easy process of synthesis, leading to improved absorption at pH 6. The N-N-N-triethylammonium chitosan and carboxymethyl chitosan were used to remove Cu (II), Ni (II), and Cr (VI) by size-enhanced ultrafiltration. The optimization study was conducted to maximize the removal of heavy metal ions from aqueous solutions and the binding ability of chitosan derivatives. Chitosan and its nanocomposites are powerful biosorbents owing to the intrinsic characteristics of their amino (-NH_2_) and hydroxyl (OH) functional groups, which make them suitable for dye removal. Synthesized ZnO-chitosan nanocomposites were effective in removing methylene blue from simulated wastewater. The excellent adsorption characteristics of chitosan nanocomposites have been highlighted by the successful adsorption of methylene blue up to six cycles (Zango et al., 2022). The *in-situ* precipitation process successfully generated ZnO/chitosan nanocomposites. The application of these nanocomposites in wastewater treatment has shown their effectiveness as a powerful adsorbent for the removal of Congo red from aqueous solutions (Nguyen et al., 2020). Table 7 represents other applications of chitosan derivatives and chitosan-based nanocomposites in wastewater treatment. In addition to the good reputation of chitosan nanocomposites in the removal of heavy metals and dyes, studies regarding antimicrobial properties and degradation efficiencies of organic and emerging contaminants are also conducted in the industry of wastewater treatment and management (Alburquenque et al., 2010; Aizat and Aziz, 2019).

**TABLE 7:** Applications of chitosan derivatives and chitosan-based nanocomposites in wastewater treatment.

Systems	Main findings	References
Chitosan-based composite hydrogels	Better adsorption capacity of methylene blue dye because of the richness of their oxygen-containing groups and their large specific surface area	Wang R et al. (2020)
Carboxymethyl chitosan-activated carbon derivatives	Effective adsorbents for the elimination of copper and lead ions from wastewater	Abdel Hafez et al. (2022)
Chitosan-based nanocomposite containing mesoporous nanosilica	Very suitable material for efficient and fast absorption of Pb(II) from an aqueous solution	Maghsoudi et al. (2021)
Thymine-containing chitosan derivative	Potential flocculant for the removal of various types of commercial pesticides from aqueous dispersions	Ghimici and Dinu (2019)
Chitosan-Cl-poly(AA)/ZrPO_4_ nanocomposite	Good remediation potential of rhodamine B dye as well as promising antibacterial behavior	Sharma et al. (2020b)
Chitosan cross-linked with 1,3-dichloroacetone	Effective product for the removal of heavy metals Pb(II), Cr(VI), Cu(II), Fe(II), and Zn(II) from poultry wastewater effluents	Atangana and Oberholster (2020)
Carboxymethyl chitosan/phytic acid composite hydrogels	Fast and stable adsorption of methyl orange and Congo red dyes from an aqueous solution	Han et al. (2021)
Metal oxides-chitosan based nanocomposites	Efficient, environmentally friendly, recyclable, and stable nanocomposites for the removal of carcinogenic polycyclic aromatic hydrocarbons from wastewater by sunlight	Rani et al. (2020)
Chitosan-lignin membranes	Environmentally friendly, inexpensive, and compostable materials demonstrating viability as a substitute for the disposal of methylene blue as a wastewater pollutant	Vedula and Yadav (2022)
Sulfonated chitosan-based flocculant	Removal of heavy metals by chelation, adsorption, and co-decantation. Materials with high flocculation performance, thermal stability, and solubility	Tang et al. (2020)

## 9 Conclusion

Chitosan represents an interesting and valuable way to valorize marine by-products. This polymer is an environmentally friendly material with several promising biological activities and specific physicochemical properties that make it the most polyfunctional and versatile of all other biopolymers. The presence of active functional groups is particularly advantageous to ensure the modification of chitosan and consequently broaden its application spectrum. Chemical and physical modification of chitosan is widely discussed to produce a variety of chitosan derivatives with high solubility and enhanced properties. However, the enzymatic route is still under development, which suggests a very bright future for the sustainable modification of chitosan. With advances in nanotechnology, chitosan-based nanocomposites are the subject of intensive research with a bright future, thanks to their unique and exceptional properties that make them more effective in many sectors. However, further research into chitosan chemistry will open up broad prospects for chitosan derivatives and chitosan in nanoparticle form and therefore offer valuable options for promising chitosan applications. The evolution of chitosan and its derivatives should be strategically oriented towards precision, responsiveness and sustainability, encompassing a broad spectrum of applications. This intentional approach positions chitosan as a key player in meeting contemporary challenges across all industries, promising a future where its diverse applications will be characterized by both efficiency and environmental responsibility.
